# Understanding the structural transformation, stability of medium-sized neutral and charged silicon clusters

**DOI:** 10.1038/srep15951

**Published:** 2015-11-03

**Authors:** Li Ping Ding, Fang Hui Zhang, Yong Sheng Zhu, Cheng Lu, Xiao Yu Kuang, Jian Lv, Peng Shao

**Affiliations:** 1 Department of Optoelectronic Science & Technology, College of Science, Shaanxi University of Science & Technology, Xian, 710021, China; 2 Department of Physics, Nanyang Normal University, Nanyang, 473061, China; 3 Institute of Atomic and Molecular Physics, Sichuan University, Chengdu, 610065, China; 4 Beijing Computational Science Research Center, Beijing, 100084, China

## Abstract

The structural and electronic properties for the global minimum structures of medium-sized neutral, anionic and cationic Si_*n*_^*μ*^ (*n* = 20–30, *μ* = 0, −1 and +1) clusters have been studied using an unbiased CALYPSO structure searching method in conjunction with first-principles calculations. A large number of low-lying isomers are optimized at the B3PW91/6-311 + G* level of theory. Harmonic vibrational analysis has been performed to assure that the optimized geometries are stable. The growth behaviors clearly indicate that a structural transition from the prolate to spherical-like geometries occurs at *n* = 26 for neutral silicon clusters, *n* = 27 for anions and *n* = 25 for cations. These results are in good agreement with the available experimental and theoretical predicted findings. In addition, no significant structural differences are observed between the neutral and cation charged silicon clusters with *n* = 20–24, both of them favor prolate structures. The HOMO-LUMO gaps and vertical ionization potential patterns indicate that Si_22_ is the most chemical stable cluster, and its dynamical stability is deeply discussed by the vibrational spectra calculations.

The experimental and theoretical studies of the atomic and molecular clusters are interesting topics since they constitute intermediate phases between individual atoms and bulk solids, which can be used to understand how the fundamental properties of materials evolve from isolated atoms or small molecules to a bulk phase^1^,^2^,^3^,^4^,^5^,^6^,^7^,^8^. The study of small clusters can help us to design better nanosystems with specific physical and chemical properties. Silicon is the most widely used material in the microelectronic industry. If current miniaturization trends continue, minimum device features will soon approach the size of atomic clusters. In this size regime, the structures and properties of materials often differ dramatically from those of the bulk. The study of the structures and properties of silicon clusters has been an extremely active area of current research. During the past two decades, a large number of experimental and theoretical studies have been carried out in this direction^4^,^6^,^7^,^8^,^9^,^10^. Much attention has been focused on understanding the structural and growth behavior of small or medium-sized silicon clusters^4^,^10^,^11^,^12^,^13^,^14^.

Several high-resolution photoelectron, Raman and infrared spectra experiments have been carried out to understand the atomic structure of small silicon clusters and showed that both Si_6_ and Si_10_ have exceptional stability^11^. Ion mobility measurements have revealed much of what is known about the growth behaviors of medium-sized silicon clusters^14^. Jarrold *et al.* have determined that anionic silicon clusters are prolate shape for *n* < 27 and become more spherical-like geometry for larger clusters^11^. However, the transition from prolate to more spherical-like geometries for cationic silicon clusters was observed in between 24 < *n* < 30^11^,^12^,^13^,^14^. Up to now, most of spherical and compact clusters have been considered as theoretical models attempting to support this measurement. A lot of works have been carried out with results not always in agreement between authors^14^,^15^,^16^,^17^,^18^,^19^,^20^. Despite the enormous progress that has been made, the true lowest-energy structures for the silicon clusters in the size range of 20 ≤ *n* ≤ 30 are still debatable. The main reasons may be as follows: (i) The procedure used in the case of small clusters is not practical for larger clusters. (ii) The predicted global minima are subtle sensitivity for the selected density functional theory, or the molecular-orbital level in the *ab initio* calculations. Moreover, the determination of the true global minimum structure is also a challenging problem, because of the much increased complexity of the potential surface as well as the exponential increase of the lowest-energy structure with the number of atoms in the cluster^21^.

In order to systematically study the structural evolution and electronic properties of silicon clusters, we here present extensive structure searches to explore the global minimum geometric structures of medium-sized neutral and charged silicon clusters in the size range of 20 ≤ *n* ≤ 30, by combining our developed CALYPSO method with the density functional theory. Our first goal of this work is to gain a fundamental understanding of the ground state geometric structures in medium-sized silicon clusters. The second one is to reexamine a number of neutral and charged low-energy isomers of Si_20_-Si_30_ that have been reported previously by experiments or density functional calculations. Thirdly, we are also motivated to explore the physical mechanism of the growth behaviors of medium-sized silicon clusters and provide relevant information for further theoretical and experimental studies. The paper is organized as follows: the computational details are described in Section 2, results are presented and discussed in Section 3, and our final conclusions are given in Section 4.

## Computational Methods

Our cluster structure prediction is based on the CALYPSO method^22^,^23^,^24^. A global version of particle swarm optimization (PSO) algorithm^25^ is implemented to utilize a fine exploration of potential energy surface for a given non-periodic system. The bond characterization matrix (BCM) technique is employed to eliminate similar structures and define the desirable local search spaces. This structure prediction method has been benchmarked on LJ clusters with sizes up to 150 atoms. High search efficiency is achieved, demonstrating the reliability of the current method. The significant feature of this method is the capability of predicting the stable structure with only the knowledge of the chemical composition. It has been successful in correctly predicting structures for various systems^24^,^25^,^26^,^27^. The evolutionary variable structure predictions of the neutral and charged Si_20–30_ clusters are performed. To seek low-lying structures, the computational process can be divided into two steps. Firstly, an unbiased global search is performed, using the CALYPSO method combined with density functional theory geometric optimization. Each generation contain 30 structures, 60% of which are generated by PSO. The others are new and will be generated randomly. We follow 50 generations to achieve the converged structure. Next, among the 1000–1500 isomers for the neutral and charged Si_*n*_ clusters, the top fifteen low-lying isomers are collected as candidates for the lowest-energy structure. Those isomers with energy difference from the lowest-lying isomer less than 3 eV are further optimized at B3PW91/6-311 + G* level of functional/basis set. All the quantum chemical calculations are performed using the Gaussian 09 program package^28^. The convergence thresholds of the maximum force, root-mean-square (RMS) force, maximum displacement of atoms, and RMS displacement are set to 0.00045, 0.0003, 0.0018, and 0.0012 a.u., respectively. The effect of the spin multiplicity is also taken into account in the geometric optimization procedure. Meanwhile, the vibrational frequency calculations are performed at the same level of theory to make sure that the structures correspond to real local minima without imaginary frequency.

To verify the reliability of our calculations, we have calculated the neutral and charged silicon dimers (Si_2_, Si_2_^–^ and Si_2_^+^) through many different functionals (HF^29^, MP2^30^, B3LYP^31^,^32^, PW91^31^,^33^, PBE^34^, B3PW91^31^,^33^,^35^, B3P86^32^ as well as CCSD(T)^36^) with 6–311 + G* basis sets. The calculated results are summarized in Table 1. From Table 1, it is found that the results of bond length (r) and vibrational frequency (ω_e_) of the three-type silicon dimers based on both B3PW91 and CCSD(T) methods are in good agreement with the experimental values^37^,^38^,^39^,^40^. While the calculated dissociation energy (D_e_) of neutral Si_2_, adiabatic electronic affinity energy (AEA) of anionic Si_2_^–^ and adiabatic ionization potential (AIP) of cation Si_2_^+^ at B3PW91 level of theory are closer to the experimental values^37^,^40^, with deviation less than 2%, 5% and 3%, respectively. So, the B3PW91/6-311 + G* has been selected as the reasonable method for silicon clusters.

## Results and Discussion

**Figure 1:**
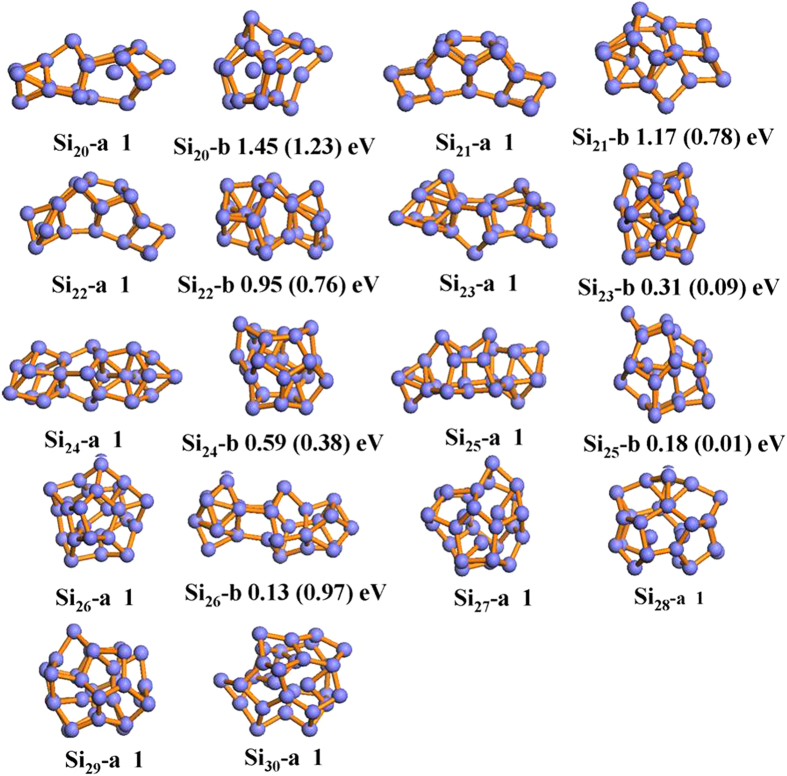
The optimized structures of neutral isomers for Si_*n*_ (*n* = 20–30) cluster at B3PW91/6-311 + G* level. The relative energies are also listed. The calculated results based on the PBEPBE/6-311 + G* level are shown in the parenthesis.

**Figure 2:**
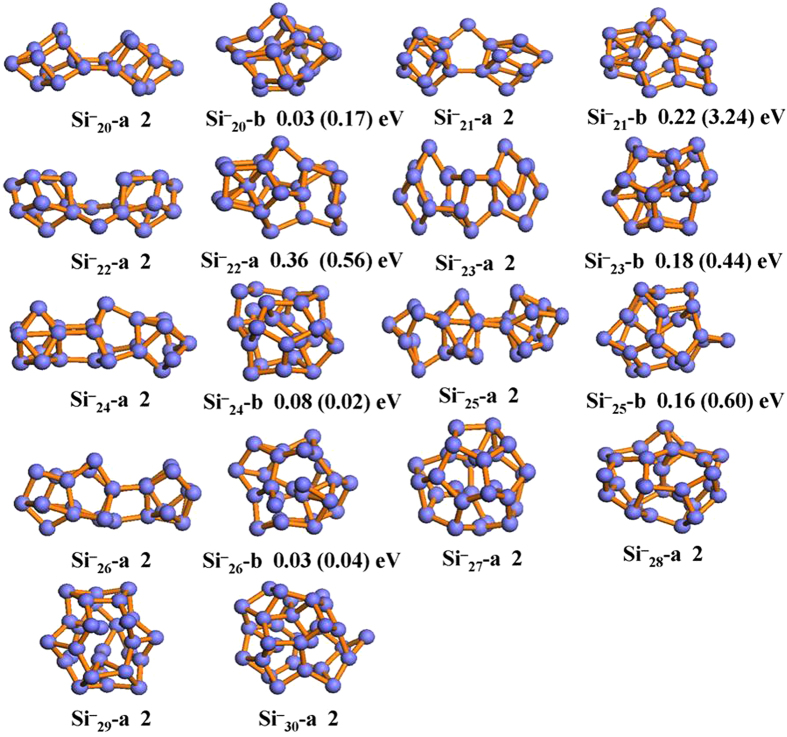
The optimized structures of anionic isomers for Si_*n*_ (*n* = 20–30) cluster at B3PW91/6-311 + G* level. The relative energies are also listed. The calculated results based on the PBEPBE/6-311 + G* level are shown in the parenthesis.

**Figure 3:**
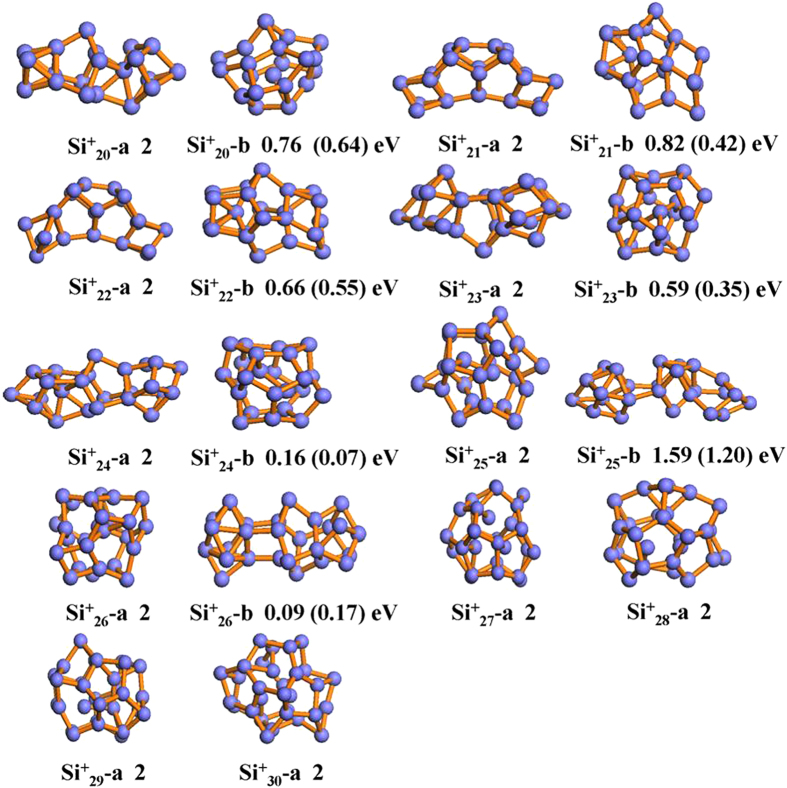
The optimized structures of cationic isomers for Si_*n*_ (*n* = 20–30) cluster at B3PW91/6-311 + G* level. The relative energies are also listed. The calculated results based on the PBEPBE/6-311 + G* level are shown in the parenthesis.

The structures found by CALYPSO searches in the range from 20 to 30 can be categorized into two kinds: prolate and spherical-like structures. All earlier known structures, experimentally and theoretically, were successfully reproduced and optimized in our current structure searches. Here, we only selected the lowest energy structures and the second low-lying isomers for neutral, anionic and cationic species and displayed them in Figs 1, 2 and 3, respectively. The other low-lying isomers of the three-type silicon clusters together with their relative energies are presented in Figures S1, S2 and S3 (see Electronic Supplementary Information). To further confirm the reliability of the present computational method, the vertical detachment energies (VDEs), adiabatic detachment energies (ADEs) and vertical ionization energies (VIPs) for large anionic and neutral silicon clusters are also calculated. The theoretical results as well as the experimental data are listed in Table 2^41^. The agreement between the experimental data and the calculated results is also excellent, which shows the accuracy of the present theoretical calculations.

### Geometric structures

In order to gain information on the growth of silicon clusters, many attempts have also been made to study the geometries of low-lying medium-sized neutral and charged silicon clusters. These include injected-ion drift-tube techniques, photoelectron spectroscopy measurements and *ab initio* calculations^11^,^18^,^42^,^43^,^44^,^45^. For neutral silicon clusters, our results indicate that the prolate structures are more stable than spherical-like structures for Si_*n*_ (20 ≤ *n* ≤ 25) clusters, then a structural transition to more spherical-like structure occurs at *n* = 26. These prolate structures can be described as stacks of stable subunits. Take the Si_20_ and Si_21_ clusters for example, their structures consist of Si_6_ unit joined by other atoms. Our result on structural transition is in agreement with the cationic mobility experiment^46^, which has shown that a structural transition from prolate to more spherical-like structures may occur in between 24 < *n* < 34. In addition, the previous theoretical studies on the medium-sized silicon clusters^6^,^47^,^48^ also indicated that the prolate structures are more favorable for Si_*n*_ (*n* = 20–26) clusters. In other words, the spherical-like isomers are expected to become more competitive energetically than the prolate isomers for larger Si_*n*_ (*n* ≥ 27) clusters.

Although considerable studies have been carried out for the neutral silicon clusters, only a few studies are available for charged clusters^11^,^41^,^48^. For anionic silicon clusters, we have examined a number of low-energy isomers which are obtained by our structural searches. Interestingly, a clear qualitative change in the geometry of these isomers is found except the silicon clusters with *n* ≥ 27. Further geometrical optimization for the final structures confirmed that the prolate Si_*n*_^−^ isomers becomes slightly more stable than spherical-like isomers and a structural transition from prolate to more spherical-like geometries occurs at *n* = 27. This observation is in complete agreement with the photoelectron spectroscopy experiments and first-principles density-functional studies by Bai *et al.*^40^. In order to gain insight into the electronic properties of the medium-sized charged silicon clusters, the vertical detachment energies (VDEs) and adiabatic detachment energies (ADEs) of Si_*n*_^−^ (*n* = 20–30) are calculated. The theoretical results are listed in Table 2 together with available experimental values^41^. It can be seen from Table 2 that the calculated VDE values of Si_*n*_^−^ (*n* = 20–30) clusters are in good agreement with experimental values, with discrepancy in the range of 0.3% to 2.7%. In addition, we also simulated the photoelectron spectra of Si_*n*_^−^ (*n* = 20–30) clusters and compared with the experiments^49^. The simulated results together with experimental photoelectron spectra are shown in Figure S4 of supplementary information. It can be seen from Figure S4, the positions and the general shape of the peaks overall agree well with experimental results. These results further give us confidence in the obtained ground-state structures for these anionic clusters. However, there is no any available experimental data to compare with our obtained ADE results for Si_*n*_^−^ (*n* = 20–30) clusters. We hope that our results for Si_*n*_^−^ (*n* = 20–30) clusters would provide more information for further investigation in the future.

Previous mobility measurement^45^ has been carried out for Si_*n*_^+^ (*n* = 20–27), which can provide information on the general shape and initial geometry of clusters. This measurement result shows that the cationic silicon clusters become spherical-like structures occurring in between 24 < *n* < 34. Based on the unbiased global search, the prolate structures (as shown in Fig. 3) are tested to be the ground state structures for Si_*n*_^+^ (*n* = 20–24) clusters. This result mirror well the shape transition observed in mobility measurement. In addition, the theoretical study^48^ on Si_*n*_^+^ (*n* = 20–27) clusters also reveals that compact Si_*n*_^+^ structures lie above the prolate for *n* ≤ 23, closely compete with them for *n* = 24 and 25, and overtake them for *n* ≥ 26 in energy. It worth mentioning that no significant difference is observed between the neutral and cation charged silicon clusters with *n* = 20–24 (see Figs 1 and 3). For Si_*n*_^+^ (*n* = 25–30) clusters, the spherical-like structures are more stable than prolate structures. These spherical-like geometries have neither the diamond-like packing of bulk silicon, nor the stuffed fullerene structure with an outer shell of pentagons and hexagons. For example, we find that the compact structure of Si_22_^+^ includes the tricapped trigonal prism (TTP) Si_9_ units which are believed to appear in the prolate structures.

Considering the structural transition point from prolate to more spherical-like geometries may relate to the chosen functional, here we re-optimized the most stable prolate structures and the next low-lying isomer with spherical-like structures for three type (neutral, anion and cation) silicon clusters at the PBE level of theory. The calculated relative energies between the most stable prolate structure and the low-lying spherical-like structure at PBE level are given in parenthesis in Figs 1, 2 and 3. It can be clearly seen that although the prolate and spherical-like structures of Si_23,25_ are almost degenerated in energy at PBE level of theory, the lowest energy structures remain unchanged for neutral, anionic and cationic silicon clusters. This suggests that the ground state structures of neutral and charged silicon clusters are independence with the used functional. Interestingly, we have also found that all the lowest-energy structures favor the low spin state.

### Relative stability

**Figure 4:**
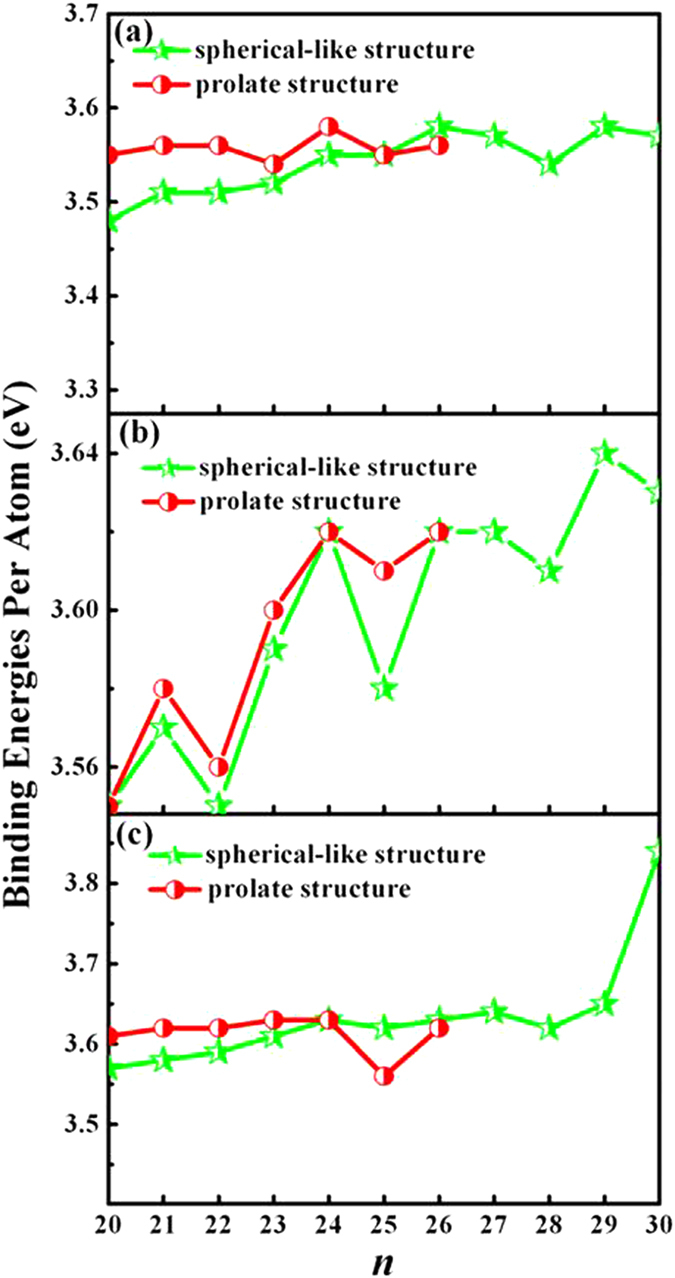
The binding energies of the spherical-like and prolate structures versus the number of atoms for neutral (**a**), anionic (**b**) and cationic (**c**) Si_*n*_ cluster, respectively.

**Figure 5:**
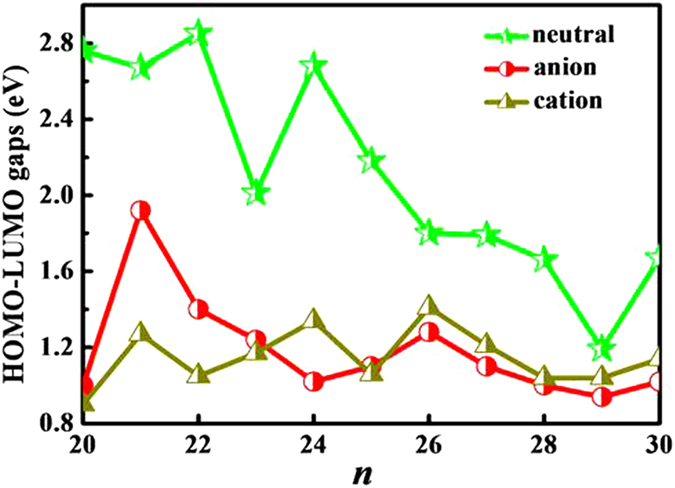
The HOMO-LUMO gaps of Si_*n*_^*μ*^ (*n* = 20–30, *μ* = 0, −1 and + 1) clusters.

It is well-known that the binding energy (E_b_) of a given cluster is a measure of its thermodynamic stability. It is defined as the difference between the energy sum of all the free atoms constituting the cluster and the total energy of the cluster. The binding energies per atom for medium-sized neutral, anionic and cationic silicon clusters (*n* = 20–30) are summarized in Table S1 of the supplementary material. Meanwhile, the binding energies as a function of cluster size *n* are plotted in Fig. 4(a–c), respectively. As is shown in Fig. 4, all the E_b_ values are not obviously lower than that of the silicon crystal (4.75 eV)^50^. In addition, the binding energies do not show a dependent behavior on the cluster size, which is in agreement with the experimental reports^16^,^17^. This shows that the structures of silicon clusters (*n* = 20–30) have different growth pattern. It can be seen from Fig. 4(a) that the binding energies of prolate structures are larger than those of spherical-like types for Si_*n*_ (*n* = 20–25), indicating that the prolate structure become more competitive energetically than the near-spherical isomers. The prolate structure of Si_25_ cluster is almost as stable as the compact structure. Furthermore, it is found that the binding energies per atom for studied silicon clusters irrespective of prolate and spherical-like structures change in a narrow region of 3.48–3.58 eV, which is also confirmed by the experiment^17^. The maximum value of 3.58 eV is found at Si_24_ with prolate structure, as well as at Si_26_ and Si_29_ clusters with spherical-like structures, which exhibits that these clusters are the most stable cluster in present study. Moreover, it is found that our calculated E_b_ (3.57 eV) of Si_30_ agree well with the previous value (3.796 eV)^19^. The E_b_ of anionic clusters as a function of cluster size *n* is displayed in Fig. 5(b). From Table S1, it is found that the spherical-like and prolate Si_20_^−^, Si_24_^−^ and Si_26_^−^ clusters are degenerate in binding energies, which can also be clearly seen from their relative energy difference as shown in Fig. 4(b). For the other clusters, the E_b_ of prolate structures are higher than those of the spherical-like structures. That is to say, the prolate structures are more energetically favorable than compact spherical-like structures. The Si_29_^−^ is the most stable structure among the obtained anionic clusters due to its largest binding energy. As for the cationic species (see Fig. 4(c)), we can clearly see that the prolate structures are more stable than the compact spherical-like structures for Si_*n*_^+^ (*n* = 20–24) clusters. This is in agreement with the result of their relative energy order. The binding energies for the prolate and compact structures increase slightly when *n* is smaller than 24. Furthermore, in the spherical-like structures, a sharply increasing is found at *n* = 29 and up to a maximum of 3.84 eV at *n* = 30. Namely, the Si_30_^+^ cluster is the most stable cluster within the cation charged clusters in the range of cluster size *n* = 20–30. The small cluster Si_20_^+^ is less stable compared with the other clusters for both prolate and spherical-like structures. From the above discussions, it is clear that the transition point from prolate structures to compact spherical-like structures occurs at *n* = 26 for neutral silicon clusters, at *n* = 27 for anions and *n* = 25 for cations. Therefore, the accepting or loss of an extra electron strongly affects the structures of silicon clusters.

The HOMO-LUMO energy gap between the highest occupied molecular orbital (HOMO) and the lowest unoccupied molecular orbital (LUMO) is a useful quantity for examining the kinetic stability. A large energy gap corresponds to a high energy required for electron excitation. The size-dependent energy gaps of the most stable Si_*n*_^*μ*^ (*n* = 20–30, *μ* = 0, −1 and +1) clusters are summarized in Table S1 and plotted in Fig. 5. By the comparison between the energy gaps of neutral, anionic and cationic silicon clusters, we can note that the curves of energy gaps for both anionic and cationic clusters have approximate tendency when *n* ≥ 25. A distinct maximum occurs at charged Si_26_^+/−^ clusters among the cluster size *n* = 25–30, indicating that Si_26_^+/−^ is relatively more chemical stable in the electronic structure compared other clusters. However, within the whole studied anionic clusters, Si_21_ˉ is the most chemical stable cluster. For neutral Si_*n*_ (*n* = 20–30) clusters, the HOMO-LUMO energy gap results reveal that the Si_22_ is the the highest kinetic stability cluster with the largest HOMO-LUMO gap of 2.85 eV.

**Figure 6:**
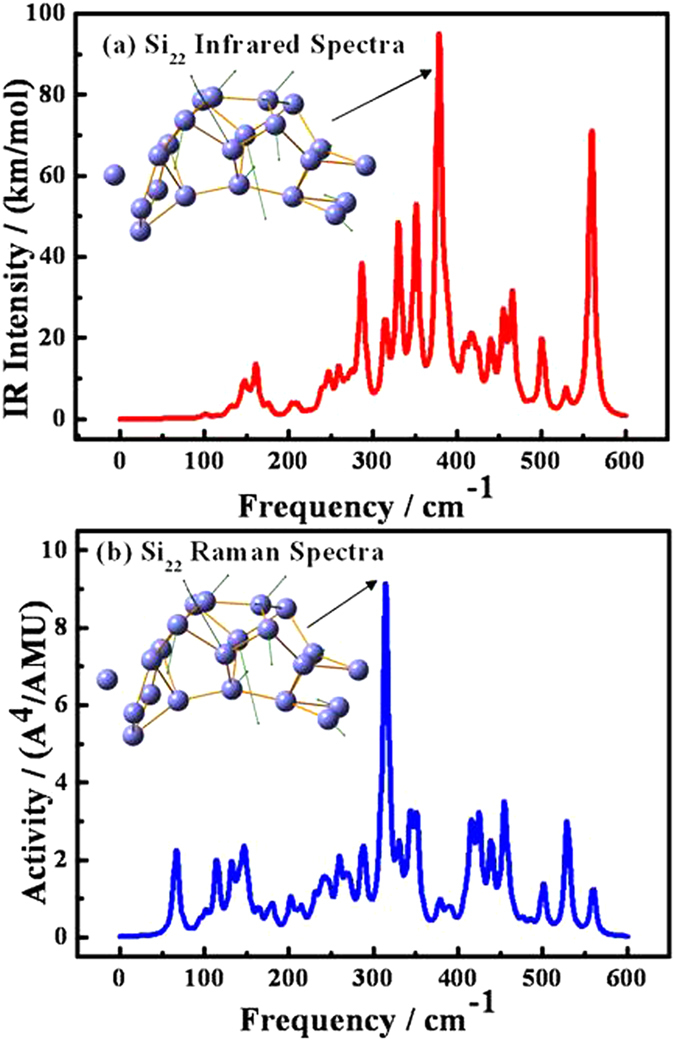
The gaussian broadened Raman activities and infrared intensities of Si_22_ cluster. Insets show the frequency modes corresponding to the highest activity or intensity.

In order to further check the dynamical stability of Si_22_, Si_21_^−^ and Si_26_^+^ clusters, we have calculated their vibrational spectra. The infrared and Raman spectra of Si_22_ are shown in Fig. 6, and the spectra of Si_21_^−^ and Si_26_^+^ clusters are shown in Figure S5 of the supporting information. We have also shown the direction of motion of the ions for the frequency with the highest Raman activity or infrared intensity. In the following, we will take the infrared and Raman spectra of Si_22_ as an example to describe the dynamical stability of the neutral silicon clusters. From the insets in Fig. 6, it can be seen that Si atom localized at the outside mainly contributes to the highest peaks of infrared spectrum and Raman activity. These spectra can provide a spectroscopic fingerprint to assist experimentalists to distinguish different species and different isomers. The infrared spectra and Raman activity of Si_22_ have several peaks due to its low C_s_-symmetry. The highest intensity peak of the infrared spectra is 95.02 km/mol, and the highest Raman activity is 9.14 Å^4^/amu. They are located at 378.00 and 314.00 cm^−1^, respectively. The complex nature of the far-infrared region when combined with the highest intensity peak of the infrared spectra can be used as a fingerprint for identifying different Si_22_ isomer. Raman activity mainly corresponds to the breathing modes, and in these modes all the ions in clusters having high symmetry move together. The high-frequency peak for Raman spectra reflects the strong bonding of cluster Si_22_.

The vertical ionization potential (VIP) is also an important parameter to assess the chemical stability of clusters. The VIP is defined as the energy differences between the total energy of neutral and cationic clusters with the same structure of the lowest-energy neutral state. Large VIPs indicate high chemical inertness. We have calculated the VIPs of the lowest-energy structures for the neutral silicon clusters. In addition, we have also calculated their adiabatic ionization potential (AIP), which is given by the formula AIP = *E*_optimized cation_ − *E*_optimized neutral_. The calculated results are listed in Table 2. As shown in Table 2, our theoretical AIPs results of Si_*n*_ (*n* = 20–30) clusters are in good agreement with the experimental data^18^. Si_22_ has the largest vertical ionization potential (7.28 eV), corresponding to its higher chemical stability. The VIP values of the other clusters are less than that of Si_22_ by 0.21 eV–1.00 eV. This suggests that Si_22_ has the higher chemical stability than others, which is in accord with the above analysis based on HOMO-LUMO gap. For Si_20_, Si_21_ and Si_25_ clusters, our calculated VIP values (7.01 eV, 7.07 eV and 6.89 eV) are in agreement with the previous theoretical results (6.76 eV^51^, 6.85 eV^44^ and 6.49 eV^44^), respectively.

### Polarizability

According to the simple perturbation theory using the one-electron wave function, the value of polarizability α can be given by the following sum-over-states (SOS) expression^52^,^53^,^54^:


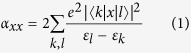


where the one electron matrix elements *l* and *k* are the antibonding (or unoccupied) and bonding (or occupied) orbitals, respectively. *ε*_*l*_−*ε*_*k*_ is the corresponding HOMO-LUMO transition energy. Accurate evaluation of matrix element is not straightforward, because it involves detailed knowledge of the wave functions of orbitals *k* and *l* as well as the relative position of each atom. Luckily, when the size of a molecule is large, the matrix element part of the equation can often be considered more or less constant. In this case, the mean polarizability per atom is then given by the invariant trace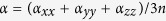, where *n* is the number of atoms in the cluster. As a benchmark test of the method, we have calculated the polarizability of isolated silicon atom. The theoretical value of 3.71 Å^3^/atom agrees well with the experimental result of 3.70 Å^3^/atom^55^. Thus, we can extend the above calculated method to the medium-sized Si_*n*_ clusters in the range of *n* = 20–30.

**Figure 7:**
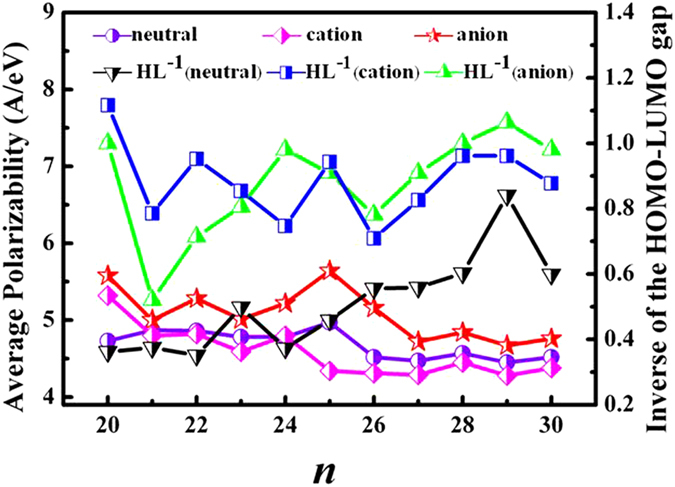
The polarizability of Si_*n*_^*μ*^ (*n* = 20–30, *μ* = 0, −1 and + 1) clusters as a function of the cluster size *n* together with the inverse of HOMO-LUMO gap.

The calculated results for Si_*n*_^*μ*^ (*n* = 20–30, *μ* = 0, −1 and +1) clusters are summarized in Table 3, in which the α values from the literature^55^ are also included for comparison. From Table 3, it can be clearly seen that the calculated α values of neutral clusters (*n* = 20–28) are in good agreement with the previous theoretical results at PBE level, with discrepancy is less than 0.25^56^. All the α values of clusters are significantly larger than the polarizability of the bulk (3.17 Å^3^/atom)^57^. In addition, the polarizabilities of Si_*n*_ clusters are not size sensitive and close to 4.60 Å^3^/atom. However, the experimental values of Schäfer *et al.*^55^ show much larger fluctuations as cluster size with an average value of about 3.5 Å^3^/atom over the range *n* = 20–28. This lack of disagreement may be explained by the temperature effects^58^. Since the average polarizability can only be directly measured in experiments if the static dipole moment of the cluster is zero. For cationic clusters, the calculated results of polarizability also show relatively small variations in the value of a over the size range 20 ≤ *n* ≤ 30, with all values significantly larger than the bulk limit. In contrast to anionic cluster, the calculated results indicate that the polarizabilities vary strongly and irregularly with size. From Fig. 7, it is interesting to note that there is a clear transition in the value of α occurring at around *n* = 27. The atomic polarizabilities can be related to the volume occupied by electrons. The compact spherical-like geometries have relatively fewer and shorter bonds, binding the valence electrons tighter with a smaller spatial volume than the prolate structures. Thus, the spherical-like clusters have smaller polarizabilities than the prolate clusters. Once again the above polarizabilities transition have demonstrated that the structure transform from prolate to spherical-like geometries in anionic silicon cluster.

To get a clear insight of the correlation between polarizability and HOMO-LUMO gap, the α values and the inverse of HOMO-LUMO gaps are plotted as a function of the cluster size *n* in Fig. 7. As is shown in Fig. 7, the curves of α values is dissimilar to the (HOMO-LUMO gap)^−1^ lines. This reveals that there is no such correlation between the polarizability and HOMO-LUMO gap among these clusters, which is consistent with the conclusions of Jackson *et al.*^59^ and Deng *et al.*^56^. For example, the neutral Si_25_, anion Si_25_^–^ and cation Si_20_^+^ have the largest polarizability (4.97, 5.64 and 5.32 Å^3^/atom, respectively) in respective species. If the inverse relationship between polarizability and HOMO-LUMO gaps is true, their (HOMO-LUMO gap)^−1^ should be the largest values. Namely, these clusters should have the smallest HOMO-LUMO gaps as well. However, that is not the case in neutral and anionic Si_25_ cluster (see Table S1). This lost correlation between the polarizability and the HOMO-LUMO gap can be attributed to the vanishing matrix element 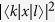
between the HOMO and LUMO in certain clusters of high symmetry. Take neutral Si_22_ cluster as an example, the wave functions of the HOMO and LUMO can be of completely different symmetry (Figure S6). This incompatibility symmetry causes that the HOMO-LUMO transition is forbidden. Consequently, all the matrix elements between these occupied and unoccupied orbitals are zero.

## Conclusions

The following conclusions emerge from the present combined CALYPSO structure searching method and density-functional theory study of medium-sized neutral, anionic and cationic silicon clusters.

- For each cluster size, an extensive search of the lowest-energy structure has been conducted by considering a number of isomers. The binding energies, HOMO-LUMO energy gaps, vertical ionization potentials, adiabatic detachment energies, polarizability including Raman activities, and infrared intensities are predicted at the B3PW91/6-311 + G* level.
- Our structural optimizations indicate that an appreciable structural transition from prolate to spherical-like geometries occur at *n* = 26 for neutral Si_*n*_ clusters, *n* = 27 for anions and *n* = 25 for cations. This is in agreement with the previous experimental observations and theoretical predictions. In addition, the growth pattern of both neutral and cationic Si_*n*_ (*n* = 20–24) clusters shows a similar behavior.
- For neutral and cationic Si_*n*_ (*n* = 20–30) clusters, the structural stabilities between prolate and spherical-like structures were further verified by binding energies. Moreover, the relative stability analysis is carried out by calculating HOMO-LUMO gaps and vertical ionization potential, which shows that Si_22_ cluster has higher stability than the neighboring clusters.
- Based on the simple perturbation theory, we have discussed the relationship between the polarizability and HOMO-LUMO gaps. The results indicate that the inverse relationship between them does not hold in general by comparing the curves of polarizability and (HOMO-LUMO gap)^−1^. How to explain this phenomenon is still an open topic.

## Additional Information

**How to cite this article**: Ding, L. P. *et al.* Understanding the structural transformation, stability of medium-sized neutral and charged silicon clusters. *Sci. Rep.* **5**, 15951; doi: 10.1038/srep15951 (2015).

## Supplementary Material

Supplementary Information

## Figures and Tables

**Table 1:** The computed values of bong length r (Å) and frequency ω (cm^−1^) for the Si_2_, Si_2_^–^ and Si_2_^+^ dimers at different levels together with their corresponding experimental values.

Method	clusters								
	Si_2_			Si_2_^–^			Si_2_^+^		
	r	ω	D_e_	r	ω	AEA	r	ω	AIP
HF	2.22	562.52	1.52	2.16	604.99	1.04	2.26	489.60	7.18
MP2	2.25	546.29	2.61	2.20	531.86	1.86	2.26	482.82	7.39
B3LYP	2.28	485.51	3.06	2.20	536.68	2.09	2.30	434.23	7.92
PW91	2.30	468.72	3.38	2.21	519.91	2.14	2.31	426.29	7.87
PBE	2.30	468.76	3.37	2.22	519.63	2.11	2.32	426.98	7.83
B3P86	2.27	498.51	3.24	2.19	550.71	2.65	2.28	451.55	8.64
**B3PW91**	**2.27**	**497.09**	**3.13**	**2.19**	**549.99**	**2.10**	**2.29**	**450.16**	**7.94**
CCSD(T)	2.26	496.93	2.52	2.20	535.97	1.70	2.28	461.55	7.42
**Exp.**	**2.25**^a^	**509 ± 10**^a^	**3.21**^b^	**2.13**^a^	**528 ± 10**^a^	**2.17**^a^	**2.27**^c^	**471.8**^c^	**7.92**^d^

In addition, the dissociation energy D_e_ (eV) for Si_2_, adiabatic electron affinity AEA (eV) for Si_2_^–^ and adiabatic ionization potential AIP (eV) for Si_2_^ + ^are also calculated.

^a^ref. 37.

^b^ref. 38.

^c^ref. 39.

^d^ref. 40.

**Table 2:** The theoretical calculated vertical detachment energy (VDE), compared to the experimental values and the previous calculated results at PBEPBE/6-311 + G* level for anions.

Cluster	VDE			ADE	Cluster	VIP	AIP	
	Thiswork	Exp.^a^	Theor.^a^				Thiswork	Exp.^b^
Si_20_^–^	3.65	3.57	3.587	1.52	Si_20_	7.06	7.05	7.46–7.53
Si_21_^–^	3.60	3.57	3.564	1.91	Si_21_	7.07	6.98	6.80–6.94
Si_22_^–^	3.43	3.37	3.299	1.16	Si_22_	7.28	6.85	5.85–5.95
Si_23_^–^	3.27	3.26	3.194	2.48	Si_23_	6.60	6.21	5.95–6.05
Si_24_^–^	3.56	3.66	3.597	2.43	Si_24_	7.03	6.81	5.95–6.05
Si_25_^–^	3.24	3.21	3.206	2.02	Si_25_	6.89	6.59	5.95–6.05
Si_26_^–^	3.27	3.34	3.311	3.17	Si_26_	6.74	6.40	5.90–5.95
Si_27_^–^	3.31	3.41	3.142	2.96	Si_27_	6.50	6.29	5.80–5.90
Si_28_^–^	3.43	3.38	3.271	3.20	Si_28_	6.56	6.38	5.80–5.90
Si_29_^–^	3.40	3.41	3.402	3.25	Si_29_	6.28	6.10	5.8
Si_30_^–^	3.51	3.46	3.613	3.27	Si_30_	6.61	6.40	5.70–5.80

The adiabatic detachment energy (ADE) for anions, as well as vertical and adiabatic ionization potential (VIP and AIP) for neutral cluster are also collected. All energies are in units of eV.

^a^ref. 41.

^b^ref. 18.

**Table 3:** The computed mean polarizability per atom of Si_*n*_^*μ*^ (*n* = 20–30, *μ* = 0, −1 and +1) clusters together with the available theoretical results.

Cluster	α		Cluster	α	Cluster	α
	Present	Literature^a^				
Si_20_	4.73	4.93	Si_20_^–^	5.58	Si_20_^+^	5.32
Si_21_	4.87	5.09	Si_21_^–^	5.00	Si_21_^+^	4.80
Si_22_	4.86	4.86	Si_22_^–^	5.28	Si_22_^+^	4.82
Si_23_	4.78	4.99	Si_23_^–^	5.01	Si_23_^+^	4.59
Si_24_	4.78	4.97	Si_24_^–^	5.22	Si_24_^+^	4.80
Si_25_	4.97	5.13	Si_25_^–^	5.64	Si_25_^+^	4.34
Si_26_	4.52	4.70	Si_26_^–^	5.16	Si_26_^+^	4.31
Si_27_	4.47	4.64	Si_27_^–^	4.72	Si_27_^+^	4.29
Si_28_	4.57	4.57	Si_28_^–^	4.84	Si_28_^+^	4.45
Si_29_	4.45		Si_29_^–^	4.67	Si_29_^+^	4.28
Si_30_	4.52		Si_30_^–^	4.76	Si_30_^+^	4.38

α is in units of Å^3^/atom.

^a^ref. 55.
