# Supplementary material for: Understanding the structural transformation, stability of medium-sized neutral and charged silicon clusters
Source: Sci Rep. 2015 Nov 3;5:15951. doi: 10.1038/srep15951 (PMC4630652; doi:10.1038/srep15951)
Supplement: Supplementary Information [file srep15951-s1.doc]

**Understanding the structural transformation, stability of medium-sized** **neutral and** **charged silicon clusters**

Li Ping Ding^1^, Fang Hui Zhang^1^, Yong Sheng Zhu^2^, Cheng Lu^2,4,*^, Xiao Yu Kuang^3,*^, Jian Lv^4,*^, and Peng Shao^1^

^1^College of Science, Shaanxi University of Science & Technology, Xian 710021, China

^2^Department of Physics, Nanyang Normal University, Nanyang 473061, China

^3^Institute of Atomic and Molecular Physics, Sichuan University, Chengdu 610065, China

^4^Beijing Computational Science Research Center, Beijing 100084, China

*Correspondence author. E-mail: [lucheng@calypso.cn (Cheng Lu), scu_kuang@163.com](mailto:lucheng@calypso.cn (Cheng Lu), scu_kuang@163.com) (Xiao-Yu Kuang), and [lvjian@calypso.cn](mailto:lvjian@calypso.cn) (Jian Lv)

| cluster | E_b_ | | HL | cluster | E_b_ | | HL | cluster | E_b_ | | HL |
| --- | --- | --- | --- | --- | --- | --- | --- | --- | --- | --- | --- |
|  | spherical | prolate |  |  | spherical | prolate |  |  | spherical | prolate |  |
| Si_20_ | 3.48 | 3.55 | 2.76 | Si_20_^–^ | 3.55 | 3.55 | 1.00 | Si_20_^+^ | 3.57 | 3.61 | 0.90 |
| Si_21_ | 3.51 | 3.56 | 2.67 | Si_21_^–^ | 3.57 | 3.58 | 1.92 | Si_21_^+^ | 3.58 | 3.62 | 1.27 |
| Si_22_ | 3.55 | 3.56 | 2.85 | Si_22_^–^ | 3.56 | 3.55 | 1.40 | Si_22_^+^ | 3.59 | 3.62 | 1.05 |
| Si_23_ | 3.56 | 3.54 | 2.01 | Si_23_^–^ | 3.59 | 3.60 | 1.24 | Si_23_^+^ | 3.61 | 3.63 | 1.17 |
| Si_24_ | 3.55 | 3.58 | 2.68 | Si_24_^–^ | 3.62 | 3.62 | 1.02 | Si_24_^+^ | 3.63 | 3.63 | 1.34 |
| Si_25_ | 3.55 | 3.55 | 2.18 | Si_25_^–^ | 3.58 | 3.61 | 1.10 | Si_25_^+^ | 3.62 | 3.56 | 1.06 |
| Si_26_ | 3.67 | 3.56 | 1.80 | Si_26_^–^ | 3.62 | 3.62 | 1.28 | Si_26_^+^ | 3.63 | 3.62 | 1.41 |
| Si_27_ | 3.57 |  | 1.79 | Si_27_^–^ | 3.62 |  | 1.10 | Si_27_^+^ | 3.64 |  | 1.21 |
| Si_28_ | 3.54 |  | 1.66 | Si_28_^–^ | 3.61 |  | 1.00 | Si_28_^+^ | 3.62 |  | 1.04 |
| Si_29_ | 3.58 |  | 1.19 | Si_29_^–^ | 3.64 |  | 0.94 | Si_29_^+^ | 3.65 |  | 1.04 |
| Si_30_ | 3.57 |  | 1.67 | Si_30_^–^ | 3.63 |  | 1.02 | Si_30_^+^ | 3.84 |  | 1.14 |

**Table S1.** The calculated average binding energy per atom (E_b_) and the HOMO-LUMO energy gaps (HL) for the lowest-energy Si*_n_^μ^* (*n* = 20-30, *μ* = 0, -1 and +1) clusters. All energies are in units of eV.

| cluster | frequency |
| --- | --- |
| Si_20_ | 333, 346, 542, 414, 381, 349 |
| Si_21_ | 350, 523, 328, 387, 388, 563 |
| Si_22_ | 560, 378, 330, 379, 286, 351 |
| Si_23_ | 521, 411, 224, 473, 385, 455 |
| Si_24_ | 493, 356, 418, 415, 210, 350 |
| Si_25_ | 356, 329, 546, 249, 251, 453 |
| Si_26_ | 465, 480, 493, 266, 446, 376 |
| Si_27_ | 315, 367, 197, 205, 407, 456 |
| Si_28_ | 125, 150, 101, 186, 331, 200 |
| Si_29_ | 134, 408, 169, 389, 323, 468 |
| Si_30_ | 383, 317, 282, 408, 494, 384 |

**Table S2.** The vibration frequencies with most IR intensities of the lowest-energy neutral Si*_n_* (*n* = 20-30) clusters.

| cluster | frequency |
| --- | --- |
| Si_20_^–^ | 193, 167, 521, 345, 197, 320 |
| Si_21_^–^ | 466, 519, 194, 122, 398, 404 |
| Si_22_^–^ | 472, 488, 236, 192, 427, 413 |
| Si_23_^–^ | 477, 198, 378, 441, 249, 404 |
| Si_24_^–^ | 502, 131, 304, 219, 142, 387 |
| Si_25_^–^ | 279, 190, 250, 386, 357, 457 |
| Si_26_^–^ | 199, 157, 445, 197, 228, 415 |
| Si_27_^–^ | 362, 313, 302, 261, 192, 198 |
| Si_28_^–^ | 48, 225, 385, 138, 178, 471 |
| Si_29_^–^ | 496, 448, 336, 76, 465, 316 |
| Si_30_^–^ | 410, 491, 387, 332, 134, 361 |

**Table S3.** The vibration frequencies with most IR intensities of the lowest-energy anionic Si*_n_*^–^ (*n* = 20-30) clusters.

| cluster | frequency |
| --- | --- |
| Si_20_^+^ | 358, 510, 412, 337, 295, 262 |
| Si_21_^+^ | 346, 322, 328, 381, 386, 352 |
| Si_22_^+^ | 87, 377, 325, 377, 105, 274 |
| Si_23_^+^ | 515, 412, 477, 474, 446, 177 |
| Si_24_^+^ | 442, 281, 85, 481, 409, 333 |
| Si_25_^+^ | 464, 455, 425, 346, 475, 392 |
| Si_26_^+^ | 481, 471, 356, 373, 451, 438 |
| Si_27_^+^ | 67, 193, 200, 365, 448, 433 |
| Si_28_^+^ | 473, 457, 451, 396, 272, 401 |
| Si_29_^+^ | 416, 494, 329, 392, 346, 136 |
| Si_30_^+^ | 311, 425, 315, 288, 286, 181 |

**Table S4.** The vibration frequencies with most IR intensities of the lowest-energy cationic Si*_n_*^+^ (*n* = 20-30) clusters.


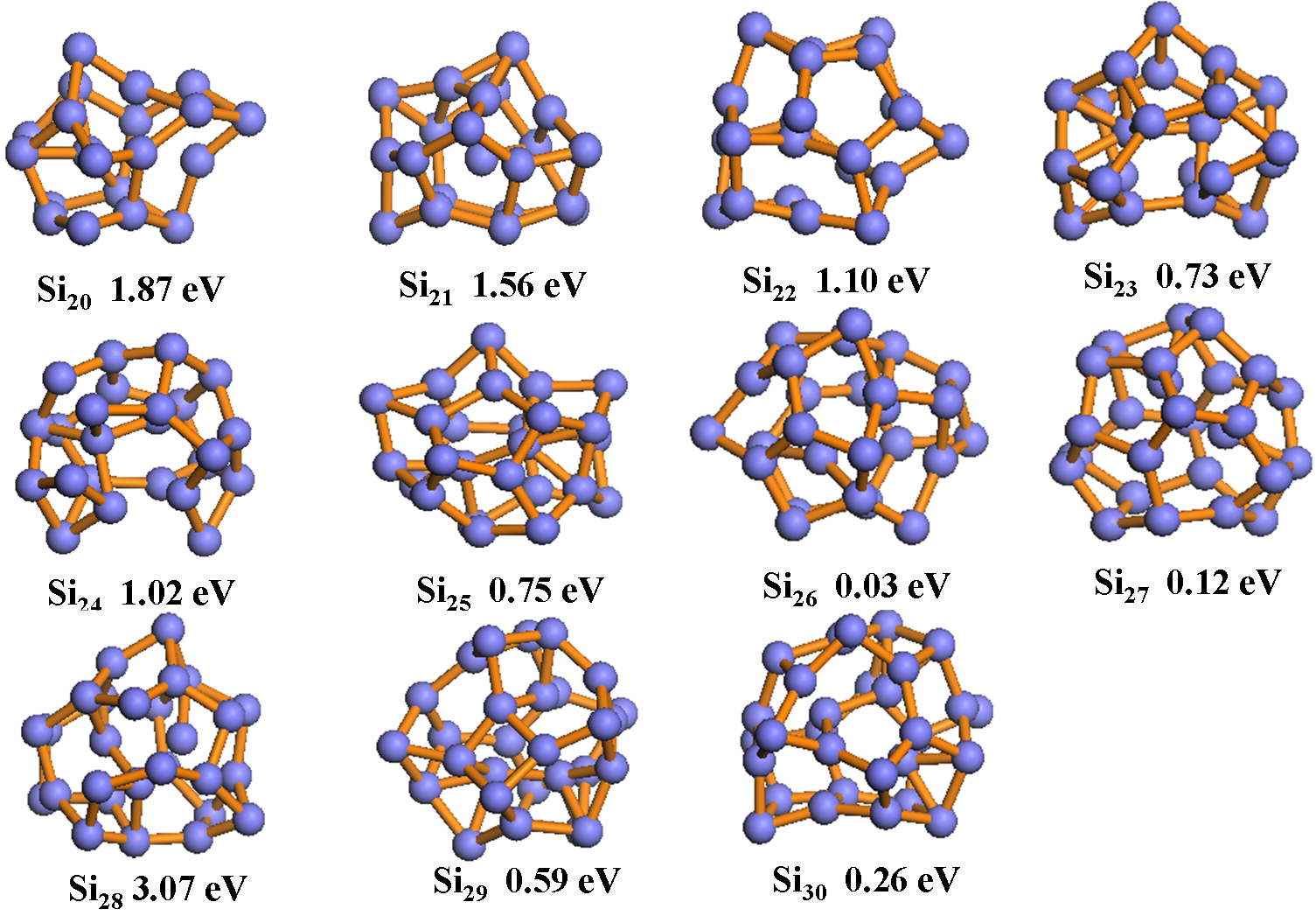


**Figure S1.** Optimized the low-lying isomer of neutral Si*_n_* (*n* = 20-30) clusters together with the relative energy (eV).


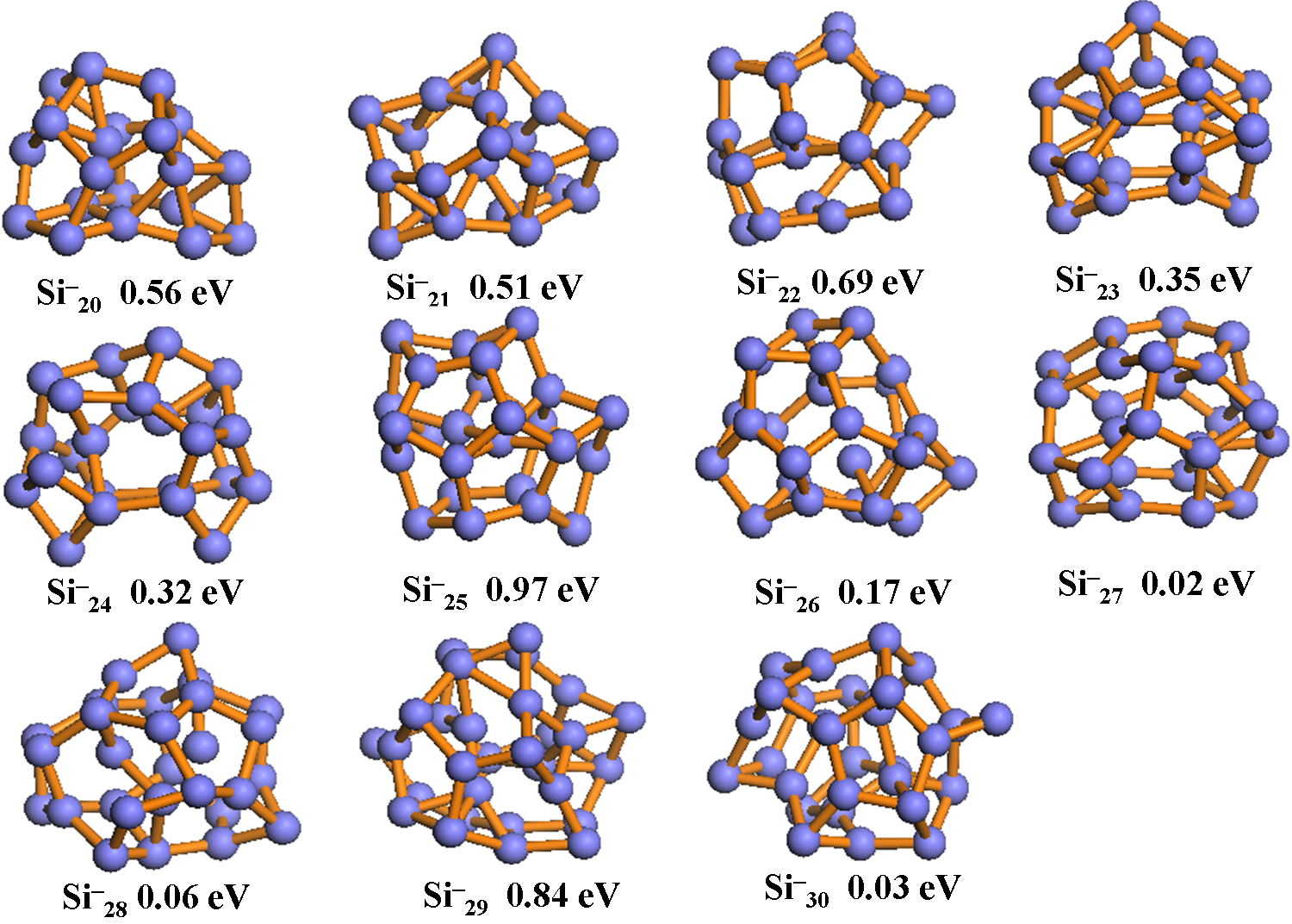


**Figure S2.** Optimized the low-lying isomer of anionic Si*_n_^–^* (*n* = 20-30) clusters together with the relative energy (eV).


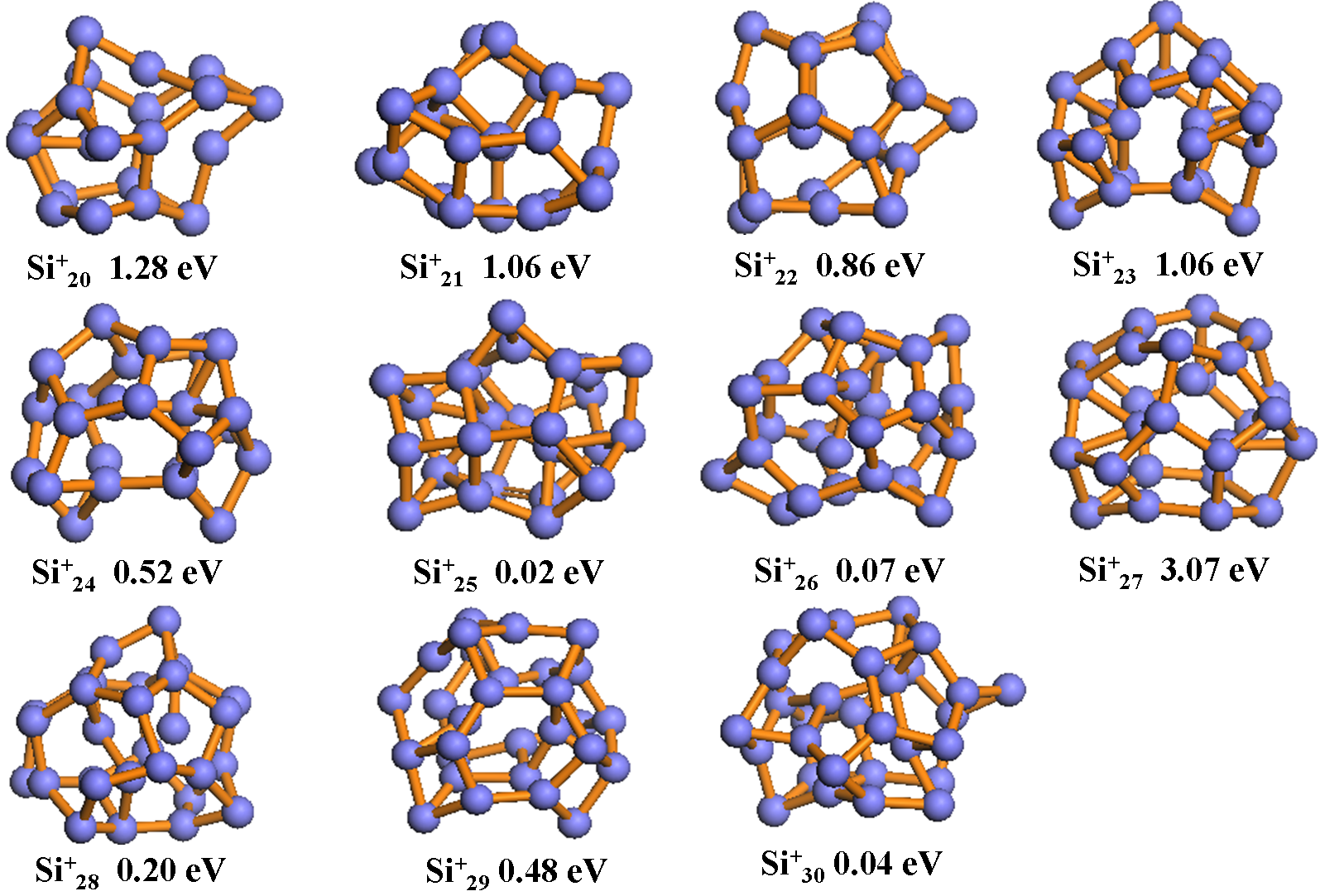


**Figure S3.** Optimized the low-lying isomer of cationic Si*_n_^+^* (*n* = 20-30) clusters together with the relative energy (eV).


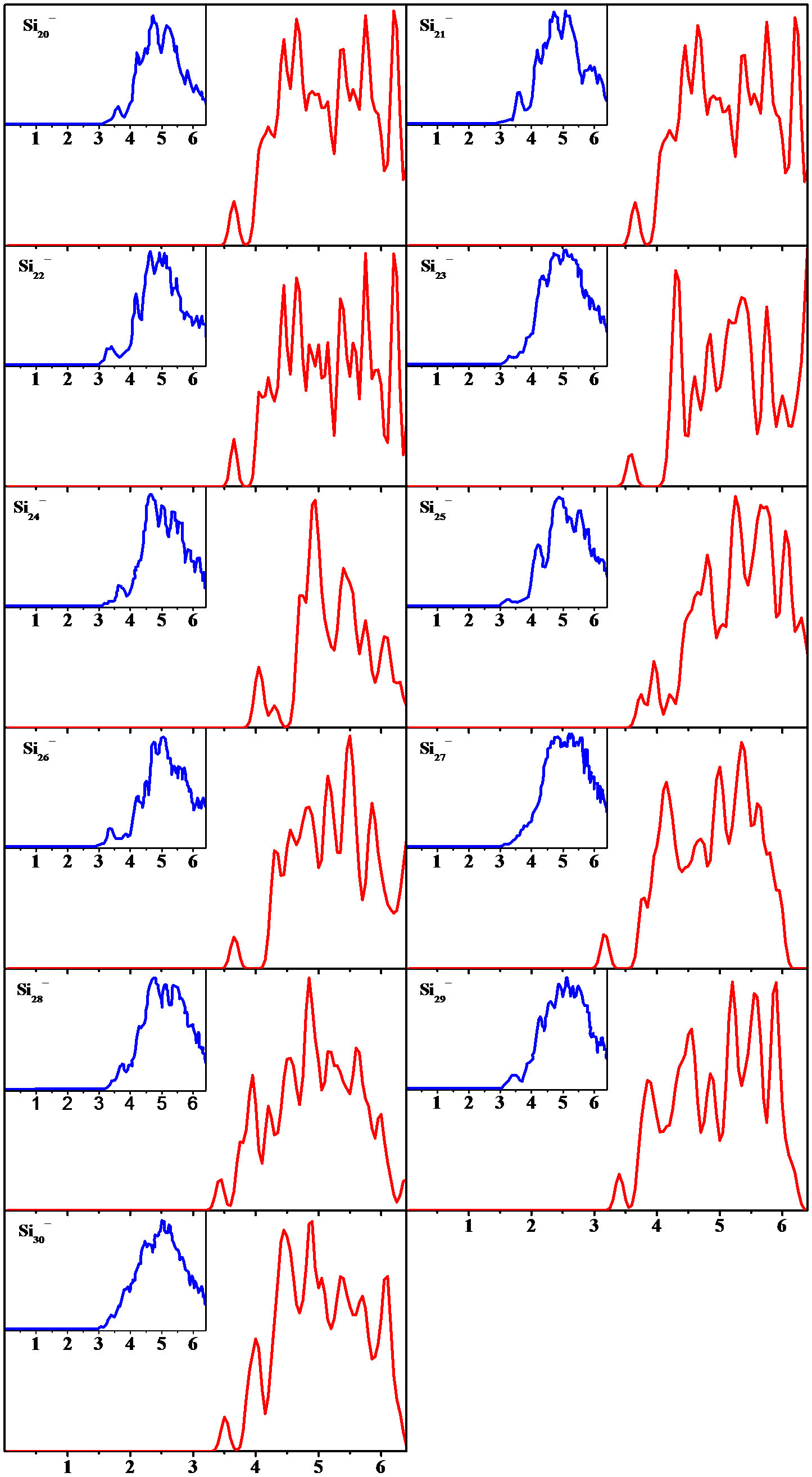


**Figure S4.** Simulated photoelectron spectra for the lowest-energy structures of Si*_n_*^—^(*n* = 20-30) clusters. The blue curves of inserted figures are experimental photoelectron spectra measured (6.4 eV photon energy).


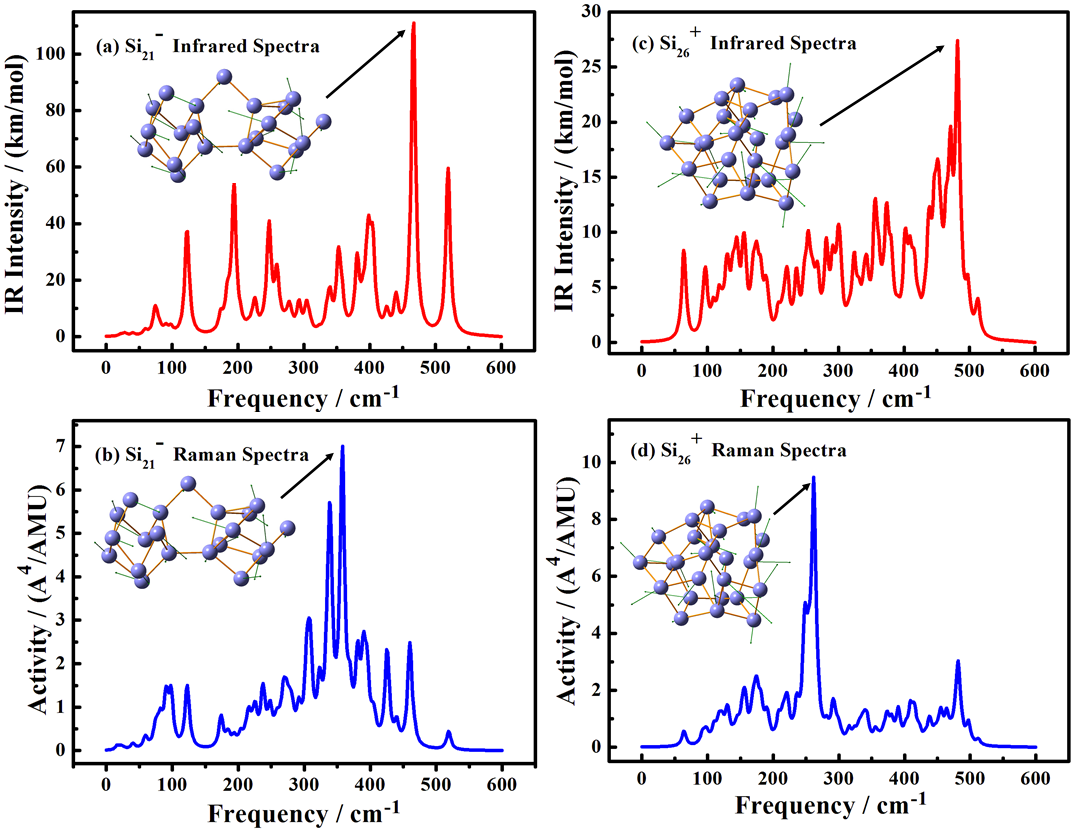


**Figure S5.** The gaussian broadened Raman activities and infrared intensities of Si_21_^¯^ and Si_26_^+^ clusters. Insets show the frequency modes corresponding to the highest activity or intensity.


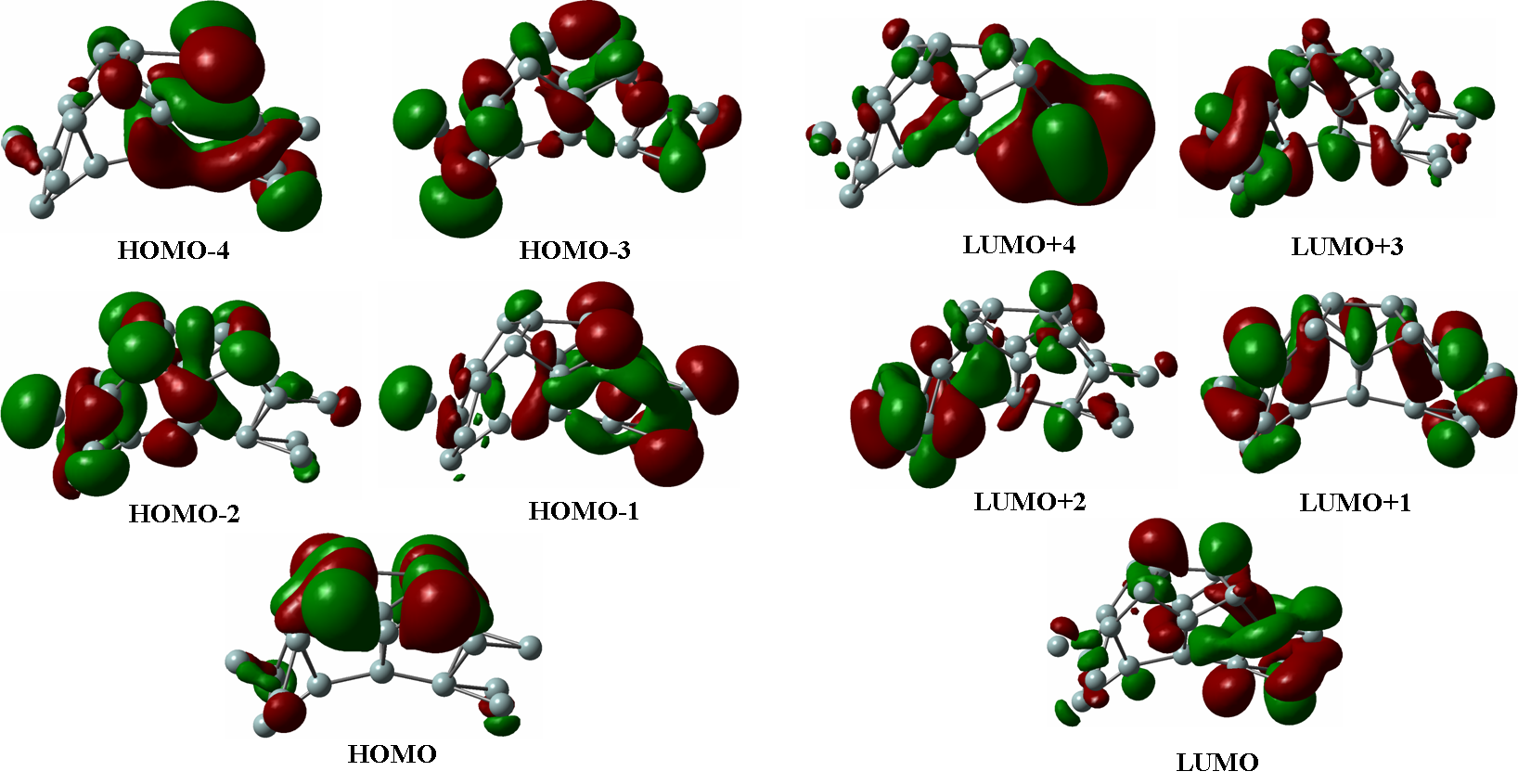


**Figure S6.** Contour plots of the wave functions for the ten consecutive frontier molecular orbitals (from LUMO+4 to HOMO-4) of Si_22_ cluster.
